# Health Care Financing Systems and Their Effectiveness: An Empirical Study of OECD Countries

**DOI:** 10.3390/ijerph16203839

**Published:** 2019-10-11

**Authors:** Viera Ivanková, Rastislav Kotulič, Jaroslav Gonos, Martin Rigelský

**Affiliations:** 1 Department of Economics and Economy, Faculty of Management, University of Prešov in Prešov, Konštantínova 16, 080 01 Prešov, Slovakia; viera.ivankova@smail.unipo.sk (V.I.); jaroslav.gonos@unipo.sk (J.G.); 2 Department of Marketing and International Trade, Faculty of Management, University of Prešov in Prešov, Konštantínova 16, 080 01 Prešov, Slovakia; martin.rigelsky@smail.unipo.sk

**Keywords:** health care financing system, health expenditure, health care effectiveness, health outcomes, OECD

## Abstract

*Background*: The primary aim of the research in the present study was to determine the effectiveness of health care in classifying health care financing systems from a sample of OECD (Organisation for Economic Co-operation and Development) countries (2012–2017). This objective was achieved through several stages of analysis, which aimed to assess the relations between and relation diversity in selected variables, determining the effectiveness of health care and the health expenditure of health care financing systems. The greatest emphasis was placed on the differences between health care financing systems that were due to the impact of health expenditure on selected health outputs, such as life expectancy at birth, perceived health status, the health care index, deaths from acute myocardial infarction and diabetes mellitus. *Methods*: Methods such as descriptive analysis, effect analysis (*η^2^*), binomial logistic regression analysis, linear regression analysis, continuity analysis (*ρ*) and correspondence analysis, were used to meet the above objectives. *Results*: Based on several stages of statistical processing, it was found that there are deviations in several of the relations between different health care funding systems in terms of their predisposition to certain areas of health outcomes. Thus, where one system proves ineffective (or its effectiveness is questionable), another system (or systems) appears to be effective. From a correspondence analysis that compared the funding system and other outputs (converted to quartiles), it was found that a national health system, covering the country as a whole, and multiple insurance funds or companies would be more effective systems. *Conclusions*: Based on the findings, it was concluded that, in analyzing issues related to health care and its effectiveness, it is appropriate to take into account the funding system (at least to verify the significance of how research premises affect the systems); otherwise, the results may be distorted.

## 1. Introduction

Health care is a wide field whose effects focus not only on the health of the population; in general, we can talk about the contribution of effective health systems to the sustainable development of the countries or to environmental management. Public health is currently one of the most discussed social themes in many countries around the world. Socio-economic disparities in health outcomes appear to be a key issue for researchers and experts [1]. There have been several studies on health effectiveness that dealt with the socio-economic factors that affect health outcomes. The evidence showed the effects of childhood poverty on health and development [2]. Fernald et al. [3] also identified the link between child development and wealth and maternal education. Other important factors that affect health outcomes include accessibility to health care [4,5,6], education [7,8,9], the number of doctors [10] and the happiness and well-being of society [11,12]. Last but not least, a significant negative impact of unemployment on self-assessed health and mental health was confirmed [13]. Poor health is widely recognized as a consequence of social disadvantage. Our study looks at the effectiveness of health care in terms of the relationship between health expenditure and health outcomes in various health care financing systems in OECD countries. The main idea of the present research arose from the assumption that increased funding must be positively reflected in a particular variable that determines the effectiveness of health care and that if systems are well designed, they should produce comparable outputs.

### Literature Review

The aim of policy-makers is to provide a level of health care comparable to that provided in Western European countries. Strengthening the health system through the implementation of health standards can be one method to significantly improve in performance and quality of health care services [14]. However, not only financial resources must be allocated to a sustainable long-term health care system. Cost effectiveness is extremely important to achieve the objectives of fiscal sustainability and access to quality health care for all citizens in a given country [15]. The policy makers of many countries have observed a fluctuation in health care spending, with the country’s health care system considerably affecting national health care spending [16]. The European Commission [17] presented the Joint Report on Health Care and Long-Term Care Systems and Fiscal Sustainability, highlighting selected key challenges for health and long-term care in the European Union (EU), as well as improving the fiscal sustainability of public health spending. According to this report, spending on health and long-term care has increased and displays a growing tendency in all EU member states. In 1990 health spending presented about 5.8% of gross domestic product (GDP), 8.7% percent in 2015 and is projected to rise to 12.6% of GDP by 2060 [18,19]. This figure is closely related to life expectancy, which, due to demographic changes and advanced technologies, will increase to 89.1 years for women and 84 years for men by 2060, resulting in a significant increase in the proportion of seniors in the total population [17]. In the relationship between expenditure and outcomes for health care, previous studies [20,21,22] reported different effects, so the causal relationship between these two variables is not entirely clear.

As health capital plays a significant role in a country’s economic growth from a long-term perspective, the health care system in a country should be supported and the World Health Organization’s recommendations on state health care spending should be considered. Investing in health care is important in terms of formation of the health capital. Health capital crucially functions in a country’s economic growth and can be identified by many indicators [23,24]. Many researchers examined the relationship of health expenditure and GDP, a bi-directional causal link between these variables was indicated. To this end, policies seek to improve the well-being of the population in the long-term by increasing health expenditure [25]. Sharma [26] investigated the relationship between health and economic growth. The results showed that the health status of the population, which is proxied by life expectancy, has a positive and significant effect on real per capita income as well as economic growth. The results reported by Boachie [27] showed that good health significantly promotes economic growth, both in the short- and long-term.

Development policy should therefore aim to increase health sector investment to improve the health conditions of the population. We can ask the question, what effective alternatives can be implemented to increase health expenditures in countries with a diverse population structure. In more developed countries, a strong positive relationship exists between national income and total health expenditure [28], but, conversely [29], in low-income countries, health care spending is less sensitive to national income changes. The increase in the national income in the country does not necessarily correspond to sufficient improvement in financing of health care. Another influential determinant of the amount of health expenditure is the demographic aging of the population [30]. Howdon and Rice [30] found that population morbidity, together with the current increase in life expectancy, exert competitive and counterbalancing pressures on health spending. Similar results have been reported by other authors [31,32] who examined the relationship between the input and output dynamics of health care systems, and showed that long-term viability has steadily increased and child mortality rates have fallen, together with rising health care spending. Van den Heuvel and Olaroiu [20] confirmed their finding that the relationship between health expenditure and health care outcomes is complex and unclear, as spending on health care affects health outcomes. For this reason, the authors stated that without careful determination of the causes influencing this relationship, the effects of increasing or reducing health care costs may lead to over- or under-estimation. Improving health care management helps to positively increase the efficiency of health spending [33].

Average life expectancy at birth is a commonly used measure of health system efficiency, economic development, and a key indicator of people’s well-being. For the management of public health resources, Cervantes et al. [34] highlighted life expectancy at birth as an indicator able to measure the performance of the health system. Health care spending for countries has heterogeneous effects on life expectancy, due to differences in population characteristics and economic factors [22]. Many studies [22,35,36] evaluated the variation in the impact of health expenditures by using linear regression, and the results showed that increasing health care spending in low life-expectancy countries can produce significant returns on the life expectancy of the population while reducing global inequalities in long-term care. Previous results [22], analyzed by quantile regression, showed rising health expenditure positively affects especially low-life countries. Bein et al. [37] also confirmed a strong and positive relationship between total health care spending and life expectancy. Contrasting results [38] have been observed among the U.S. population, where health care spending increases and life expectancy are lower than in most industrialized countries. Studies have confirmed the significant relationship between social expenditure and life expectancy. Reynolds and Avendano [38] stated that increasing social expenditure positively affects life expectancy and this positive relationship is stronger than the relationship between health expenditure and life expectancy. By testing, they found that maximum spending on education and incapacity can increase life expectancy to 80.12 years. Other U.S. research [39] examined differences between health expenditure and social expenditure and assessed their association with health indicators. The authors [39] stated that there is a significant relationship between health expenditure and low birth weight and maternal mortality, social expenditure was significantly associated with life expectancy, infant mortality, and increased potential life years lost.

Perceived health status is the most frequently applied measure of health in empirical research [40,41]. Several authors investigated the relationship between health expenditure and self-assessed health status [42], and the limited effect of expenditure was confirmed [43]. Another measure of health care effectiveness is mortality. Heijink et al. [44] stated that countries with an above-average expenditure growth showed above-average reductions in avoidable mortality. The findings of other study showed that, between 2011 and 2014, health expenditure in Italy decreased, compared to previous years, and the overall mortality increased [45]. The results of other studies [46,47] also suggested that reduced health expenditure is related to increased population mortality. Among the health outcomes that are affected by health and social expenditure, Bradley et al. [48] also identified mortality rates for lung cancer and acute myocardial infarction. On the other hand, based on data on acute myocardial infarction, Moscone et al. [49] found that health expenditure had a little impact on health outcomes, but the authors highlighted the importance of how the health expenditure is spent. Cost-effective health care could improve the outcomes of patients with acute myocardial infarction [50], which means improving health outcomes. The coexistence of diabetes in people with acute myocardial infarction is common. Zhou et al. [51] confirmed that excess expenditure associated with diabetes was substantial for people with acute myocardial infarction (AMI) or who had had an acute ischemic stroke (AIS), and they highlighted the need for both the prevention and better management of diabetes among AMI and AIS patients, which in turn may lower the financial burden of treating these conditions. At the global level, there has been an evident increase in the cost of diabetes [52], which imposes a large economic burden on health care systems across the world [53,54]. Jonsson [55] determined the relations of diabetes with economic output, indicating that diabetes accounts for 2–3% of the total health care budget in every country. Therefore, an increase in diabetes incidence and prevalence translates into a significant economic impact. The evidence revealed that the growth of expenditure relates to the growing prevalence of diabetes, and the excess cost associated with the incidence of treated type 2 diabetes increases by an average of 4% per year in France. This excess cost is mainly due to primary care expenditure, which is essentially related to drugs, nursing care, and medical devices and services [56]. Schofield et al. [57] also demonstrated that the indirect costs of diabetes through lost productive life years are considerable, both at the individual and national level. Studies on the direction of the relationship between health expenditure and health care quality are inconsistent. Most studies revealed that the association between expenditure and quality is small to moderate, regardless of whether the direction is positive or negative [58].

According to Ibrahim and Daneshvar [59], reduction in health expenditure does not necessarily reduce efficiency of health care system if the operational and technical aspects are improved. Another study [60] reported that information and communication technologies can improve the level of health care services. Stefko et al. [61] identified and quantified the impact of medical technologies (magnetic resonance devices, computed tomography devices) on the efficiency of the health care facilities. The authors of other study [62] detected differences in the performance of the health care systems. These differences reflect various co-innovations and technologies given the technology available.

The relationship between expenditure on health and life expectancy and the health status of the population points to the existence of significant differences between countries and suggests that health policies should focus on reducing health inequalities among countries in the EU. A deeper understanding of the determinants of public spending on health care may lead to more effective design of efficient health policies [31,63].

## 2. Materials and Methods

### 2.1. Aim of Research

The primary objective of the research in the present study was to determine the effectiveness of health care in classifying health care financing systems from a sample of OECD countries over the period 2012–2017. The main focus of this study was to determine the relationships between health expenditure, considered as the percentage of a country’s GDP (Expend_H), and selected determinants of health effectiveness: average life expectancy at birth (Life_expect), perceived health status (Self_rep_H), the health care index (HC_index), deaths due to acute myocardial infarction (AMId) per 100,000 persons, and deaths due to diabetes mellitus (DIAd) per 100,000 persons from a sample of OECD countries with respect to the type of health care financing system. In addition to the aforementioned primary focus, the relationships between individual systems (Multiple Insurance Funds or Companies—MI, the National Health Care System—NHS, and the Single-Payer Model—SPM) and selected variables that determine the effectiveness and financing of health care were also assessed.

Based on the above, a research question was formulated:

RQ: Do health care financing systems show differences in terms of the effectiveness of health care provision?

Based on the research presented in the previous chapter, this research effort will aim to verify the following hypotheses:

H1: It is assumed that in selected financing systems, health expenditure has a significant impact on life expectancy at birth.

H2: It is assumed that in selected financing systems, health expenditure has a significant impact on perceived health status.

H3: It is assumed that in selected financing systems, health expenditure has a significant impact on the health care index.

H4: It is assumed that in selected financing systems, health expenditure has a significant impact on deaths from acute myocardial infarction.

H5: It is assumed that in selected financing systems, health expenditure has a significant impact on deaths from diabetes mellitus.

### 2.2. Description of the Sample

The sample included all countries of the OECD, including Latvia, which was officially accepted as a member on 5 July 2018 [64], and Australia, Austria, Belgium, Canada, Chile, Czech Republic, Denmark, Estonia, Finland, France, Germany, Greece, Hungary, the Netherlands, New Zealand, Norway, Poland, Portugal, Slovak Republic, Slovenia, Spain, Switzerland, Turkey, United Kingdom, and the United States. As Sweden has a different system of financing (local health systems that serve distinct geographic regions), we excluded it from the analysis.

The first variable entered into the analysis was total health expenditure (Expend_H) expressed as a percentage of GDP from the OECD [65] database for the period 2012–2017. Expend_H measures the final consumption of health care goods and services (i.e., current health expenditure), including personal health care (curative care, rehabilitative care, long-term care, ancillary services, and medical goods) and collective services (prevention and public health services as well as health administration), but excluding spending on investments. Health care is financed through a mix of financing arrangements including government spending and compulsory health insurance (Government/compulsory) as well as voluntary health insurance and private funds such as households’ out-of-pocket payments, non-government organizations (NGOs) and private corporations (Voluntary). This indicator is presented as a total and by type of financing (Government/compulsory, Voluntary, or Out-of-pocket) and is measured as a share of GDP, as a share of total health spending and in USD per capita (using economy-wide public private partnerships) [65].

The second variable entering the analysis, Life_expect, is an average life expectancy at birth from the OECD [66] databases for the period 2012–2017. Life_expect is defined as how long, on average, a newborn can expect to live if current death rates do not change. However, the actual age-specific death rate of any particular birth cohort cannot be known in advance. If rates are falling, actual life spans will be higher than life expectancy calculated using current death rates. Life_expect is one of the most frequently used health status indicators. Gains in Life_expect can be attributed to a number of factors, including rising living standards, improved lifestyle, better education, and greater access to quality health services. This indicator is presented as a total, by sex, and is measured in years [66].

The third variable, Self_rep_H, represents the percentage of the population that provided a positive response (good or very good) on the five-part scale (very good, good, fair, bad, or very bad) to the question “How is your health in general?”. This variable was obtained from OECD [67] databases for the period 2012–2017.

The fourth variable in the research is the variable defining health care, HC_index. This was obtained from the NUMBEO [68] database (2012–2017) and is an aggregated index of an online survey. Seven satisfaction-oriented variables are included in the index (skill and competency of medical staff, speed in completing examination and reports, equipment for modern diagnosis and treatment, accuracy and completeness in filling out reports, friendliness and courtesy of the staff, responsiveness (waiting time) in medical institutions, and convenience of location for you). The output ranges from 0 to 100.

The fifth variable, AMId, represents deaths from acute myocardial infarction per 100,000 people. This variable was obtained from OECD [67] databases for the period 2012–2017. Myocardial infarction is defined by the demonstration of myocardial cell necrosis due to significant and sustained ischaemia. It is usually, but not always, an acute manifestation of atherosclerosis-related coronary heart disease [69].

The sixth variable that is considered in the analysis, DIAd, represents deaths from diabetes mellitus per 100,000 people. This variable was obtained from OECD [67] databases for the period 2012–2017. Diabetes mellitus describes a metabolic disorder of multiple aetiology, characterized by chronic hyperglycaemia, with carbohydrate, fat and protein metabolism disturbances, resulting from defects in insulin secretion, insulin action, or both. The effects of diabetes mellitus include the long-term damage, dysfunction and failure of various organs [70].

One more variable, and hence the determinant of financing of the health care system, HC_System, was included in the analyses. Among the selected OECD countries, three systems of financing are recognized: (1) A national health care system covering the country as a whole (NHS), (2) a single health insurance fund (single-payer model; SPM) and (3) multiple insurance funds or companies (MI) [71]. Stated information was gathered from the Health Systems Characteristics Survey conducted by the OECD in 2012 and 2016. Latvia was not yet a member of the OECD, so the information was obtained from the Latvian health care website [72].

Table 1 provides the input data (based on mean) for variables such as country, health care financing system (HC_System), total health expenditure as a percentage of GDP (Expend_H), average life expectancy at birth (Life_expect), perceived health status (Self_rep_H), health care index (HC_index), deaths from acute myocardial infarction per 100,000 people (AMId), and deaths from diabetes mellitus per 100,000 people (DIAd) for the period 2012–2017.

### 2.3. Analytical Process Description

In the first step of the analysis, a descriptive analysis was used to process collected health outputs to provide information about the achieved values of the countries as a whole, but also in classifying HC_System. The descriptive analysis provided the number of countries (*N*), the arithmetic mean (*M*), and the standard deviation (*σ*) of health care outputs. This was followed by an analysis of the association between health care financing systems and selected indicators of health care effectiveness (effect size). The coefficient, *η^2^*, was used for this analysis. The output of this analysis provides information regarding whether there is a relationship between HC_System and a certain effectiveness indicator. This was followed by a logistic binomial regression analysis, the results of which show that a change in the value of a particular health variable indicates a change in the probability of acquiring a particular HC_System. The application of this analysis was determined by the MCE (misclassification error), the Null and Residual Deviance and the Hosmer and Lemeshow GOF test. The dataset was divided, according to a dummy variable of the health system, into training (80%) and testing (20%) datasets, where the confusion matrix and then the misclassification error were calculated. A lower value represents a more positive result, and the greatest emphasis should be placed on the difference between the MCE training and testing data. Additionally, the difference between the null and residual deviance should be determined, so that the result will be more positive for the model itself (the more relevant model), when the difference is smaller. The Hoslem test is suitable for analyzing the *p*-value. If the *p*-value exceeds the rate of 0.05, the model is considered to fit well. The following part of these analyses was devoted to expressing the impact of Expend_H on the selected variables of health care effectiveness. Regression analysis (Simple linear regression, Least Trimmed Squares Robust (High Breakdown) Regression, and HC3 (Heteroskedasticity-Consistent standard error estimator) robust base regression) was used for this purpose. The selection of a particular model, as well as the evaluation of the model conditions, was carried out with the help of an F test for the individual effects (refuting the effect of time in years), a stationary—Augmented Dickey-Fuller Test, an outlier—Bonferroni Outlier Test; a heteroscedasticity—Breusch-Pagan Test and the determination coefficient *R^2^*. Correlation analysis (Spearman *ρ*) was also included in this part of the analyses, completing the information of linking the various variables of health care effectiveness in the classification of health care financing systems, as well as the overall information. The last part of the analyses considered the outputs of the correspondence analysis, aiming to assess the tightness of the interconnection between health care financing systems and the selected health care effectiveness variables, which were converted into quartiles. The analysis was carried out using the Eurlidian distance, and its objectivity was assessed on the basis of the *χ^2^* coefficient and correlation (Pearson *π*). Analysis data were processed in MS Excel (Microsoft, Redmond, WA, USA) and using the RStudio (RStudio, Inc., Boston, MA, U.S.) application.

## 3. Results

This section discusses the analytical processes determining the accuracy of our established research questions. As described in the methodology, the first part of the analyses shows descriptive statistics on the variables of health care effectiveness and the variable of the total health care financing, but also on the classification of individual systems of health care financing. The following section is devoted to determining the relation between the selected health care effectiveness variables and the variable representing Expend_H within individual HC_System. The analytical processing of the issue involves expressing the impact of Expend_H on the selected health care effectiveness variables, as well as a secondary determination of the relation of the selected variables to each other. These procedures are carried out through the classification of health care financing systems in order to understand the differences between these systems. These analyses are followed by a final section that presents the correspondence analysis.

### 3.1. Descriptive Analysis

This section consists of a descriptive analysis, an assessment of the effect size of the health care financing system’s on given health outputs, analysis of relationships, and correspondence analysis visualizations. Table 2 provides the results of the calculation of the descriptive statistics.

Table 2 shows the mean (*M*), the standard deviation (*σ*), and the frequency (*N*) of the selected indicators Expend_H, Life_expect, Self_rep_H, HC_index, AMId and DIAd providing an overview of the (total) outputs achieved by all OECD countries as well as the second-degree ranking in the individual HC_System, i.e., NHS, SPM, and MI. The highest average Expend_H was found under the MI financing system. MI had an average Expend_H of 9.72% of GDP, which is 1% more than the average Expend_H in all OECD countries. For Life_expect, we noted that in countries applying the NHS model, Life_expect was the longest, averaging 80.80 years. The shortest Life_expect was in countries with SPM, averaging 79.65 years. For Self_rep_H, countries applying NHS had the highest proportion of the population assessing their health positively, i.e., either good and very good. Examining the HC_index values, Table 2 shows that the countries applying the MI model had the highest average value. The highest mortality rates for AMId and DIAd are recorded by countries applying the MI system. Based on these variables, countries applying the SPM model achieve sub-average values not only compared with other models but also in comparison with the average outputs of the OECD countries as a whole. Possible limitations may be the specificity of the countries themselves, their characteristics, performance, level of human development, macro-environment and micro-environment, aspects the social system, and education—the effects of other dimensions particular to specific countries.

### 3.2. Relation between Health Care Financing Systems and Selected Health Indicators

Table 3 shows the effect size of the nominal (qualitative) variable HC_System on selected health care indicators. The table shows how HC_System affects Expend_H, Life_expect, Self_rep_H, HC_index, AMId and DIAd. In the Life_expect, Self_rep_H, HC_index and DIAd indicators, we identified a small but not negligible effect rate. In the case of Expend_H, this was a moderate effect. AMId it was negligible rate [73].

Table 3 shows information on the possibility of a certain relation between health variables and health care financing systems. In the next step, the impact of these health variables on the likelihood of the appearance of a given HC_System is assessed.

From the results presented in Table 4, it was decided that the Hosmer and Lemeshow goodness of fit test, which includes the H_0_: a model is fit well, will be conducted first. Since the value of *p* in models 1 and 2 is lower, it is reasonable to lean towards the alternative of the statistical hypothesis, mentioned above. For this study, this means that the model has some flaws. The Deviance principle states that differences between Null and Residual values should be as small as possible, but Model 1 and 2 show higher values. The misclassification error (MCE) should be as small as possible, and although the measured values are higher, they are still acceptable. The regression model was based on a modern methodology of comparing training (80%) and testing (20%) data. The MCE should be as small as possible. The above values can be assessed, and the most positive result was found for model 3. The collinearity (VIF) of these models was also assessed, where for Model 1, the highest measured rate was that of Expend_H, which was approximately 2.72; for Model 2, the highest measured rate was that of Life_expect, which was 2.11; and for Model 3, the highest measured VIF was that of Life_expect, which was approximately 2.06. Based on this information, it was found that the degree of collinearity in the models was acceptable. 

As mentioned, the models in question are not ideal, but they are sufficient to describe the effects of the selected variables. Table 5 shows the output of the models. Based on the results in Table 5, it was decided to first assess the significance of the impact. If the impact is significant, it is appropriate to assess the coefficients. The coefficients are evaluated firstly through their polarity and secondly through their absolute value. If the polarity is positive, e.g., with the MI system for Expend_H, it is possible to say that with increasing Expend_H, there is a significant increase in the likelihood that the system will be an MI and not another system (NHS, SPM). As can be correctly concluded, certain health variables tend to be inclined toward specific funding systems. It can therefore be assumed that, in certain funding systems, there will be different effectiveness in terms of Expend_H. The following sections of the study address this assumption.

### 3.3. Impact of Health Expenditure on Selected Health Care Indicators

Table 6 shows the outcomes of the whole process of selecting the most appropriate mechanism for determining the impact of Expend_H on the selected health care indicators. Since the time series (2012–2017) was entered into the data, a test was applied to determine the suitability of the panel model of regression analysis (fixed effect), if the *p-*value is greater than 0.05, and the classical OLS model (H_0_: the panel variable has a significant effect) seems to be more suitable. As can be seen, the reported *p-*value is, in all cases, almost equal to one. While there was only a small time series, the stationarity was tested (H_0_: the series has a unit root (i.e., non-stationary)). As is evident, there is a stationary series in all cases, so time does not need to be taken into account. The outlier defines the *p-*value of the significance of the first most significant outlier (H_0_: the outlier is not significant). The decision concerning presence was also supported by the analysis of the model visualizations, which are not mentioned here. Based on the Gauss-Marks theorem, the heteroscedasticity (H_0_: the constant residual variance) for BLUE (the Best Linear Unbiasted Estimate) is an extremely important element. In models in which significant heteroscedasticity occurred, the coefficients were derived using the HC3 estimator. The Reg. Model column points to the most suitable model, OLS is the simple linear regression Ordination Least-Squares; OLS HC3 is the OLS with the HC3 estimator; and LTS is the Least Trimmed Squares Robust (High Breakdown) Regression. The *R^2^* coefficient of determination, which is shown in the last column, represents a measure of what portion of the percentage of the variability of the dependent variable is explained by the independent variable, and a suitable rate is greater than approximately 30% (0.3). Table 7 determines the statistical significance of the models and the rate of the coefficients affecting the dependent variable. This output can be seen as one of the most valuable in terms of assessing the ideas of this study.

First of all, it is advisable to focus on the probability of the models (Sig.), and the zero statistical hypothesis (H_0_: the impact is not significant) is rejected for most models. In view of the assumptions presented in the section describing the methodology, it is appropriate to point out that each of the formed hypotheses (H1–H5) has been confirmed, so the effect of Expend_H on health care effectiveness outputs is evident for the selected health care systems. Values of *p* less than 0.05 are highlighted. A very important indicator of the level of impact is the coefficient, *β_1_*, which plays an irreplaceable function in comparing the impact of different HC_System. The highest value of the coefficient, *β_1_*, between health care systems is highlighted. In interpreting and determining the impact differences, i.e., the effectiveness of health care, the coefficient of determination must also be taken into account. In the Life_expect models, the NHS system (*R^2^* = 0.5716) appears to be the most effective, and the other two systems show lower coefficients and *R^2^* values, which could easily be challenged. The recommendation is to perceive other outputs in this way as well. At this moment, it is clear, from the above, that not every funding system declares the same outputs in relation to the impact of funding on health outcomes. The NHS funding model even appears to be ineffective in Model 10. These outputs indicate some system weaknesses.

Table 8 shows the forecast outputs for the value of 8.72, which represents the total average rate of Expend_H (%), as a percentage of a country’s GDP. More positive predictive outputs of systems are highlighted. Attention should be paid to the fact that the diseases that are frequent in developed countries (AMId, DIAd) were not, as expected, significant impacted by expenditure. It is also possible to deduce, from the above, the shortcomings of the systems, which are identifiable in variables where relations have not been confirmed, meaning that the models showed a relatively low *R^2^* value. For example, if the NHS model is taken into consideration, the relation between Expend_H and AMId or DIAd did not have to be confirmed, as the population ages, and thus the incidence of these diseases becomes more frequent. If these connections were to be considered in an exact way, it is necessary to know the extent to which the individual variables are related to each other, and this information is provided in Table 9.

Table 9 is divided into four sections: the first three determine the link between the individual systems of health care financing, and the last section shows the overall correlation. Above the diagonal, the measure of the relationship (Spearman *ρ*) is shown, and below the diagonal, the value of *p* is shown. Secondly, health care systems can be read and compared in a simplified way in terms of expenditure effectiveness (Expend_H).

### 3.4. Correspondence Analysis

In the correspondence analysis application, we grouped the OECD countries into four quartiles: Q1, Q2, Q3, and Q4. The lower quartile Q1 (<Q1) represents 25% of OECD countries with the lowest results for the surveyed variable. Conversely, the top quartile Q4 (>Q3) contains 25% of OECD countries with the highest values for the surveyed variable. Table 10 shows that countries such as Australia (NHS), Israel (MI), and Norway (NHS) are countries with the most promising results of Life_expect, Self_rep_H and HC_index. Other countries with favorable values include Austria (MI), Luxembourg (SPM), New Zaeland (NHS) and Spain (NHS). The countries that achieve the least attractive values of the examined variables are Hungary (SPM), Latvia (NHS), Poland (SPM) and Turkey (SPM).

Table 11 quantifies the basic outputs of the correspondence analysis for the selected variables in relation to the HC_System. The greatest importance can be attributed to the sig. line, which displays information in the statistical significance of linking specific variables. As is clear in the case of AMId, this is not a significant relation. The last line provides an idea of the relations between the analyzed variables. The minimum acceptable value may be considered to be 0.2. The eigenvalue determines the degree of the importance of dimensions.

**Figure 1:**
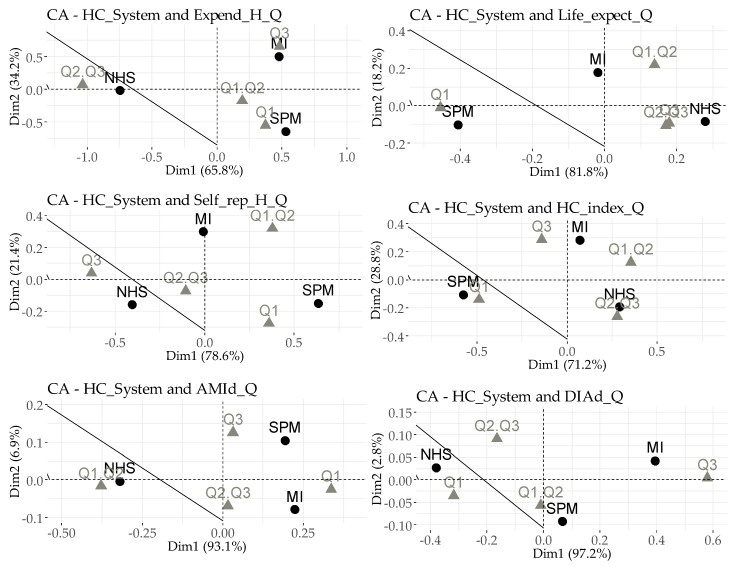
Health expenditure (Expend_H) and indicators of health care effectiveness compared with health care financing system (HC_System).

The most significant outputs of this section are the links between the categories (quartiles) of the selected health variables and the individual HC_System (MI, NHS, SPM), as shown in Figure 1. The basic rule is that the closer the categories are to each other, and at the same time the further away from other categories of the analyzed variables, the more significant the output. When interpreting Figure 1, it is necessary to take into account the scale of axes X (Dim1) and Y (Dim2), as not all graphs have the same scales.

Focusing on linking the funding system to health expenditure (Expend_H_Q), the closest link that can be identified is that between MI and Q3, which can be interpreted as the highest expenditure in this system, so one could expect that the effectiveness of the health care in system MI will be the highest. In terms of funding, the NHS has the second highest expenditure, and the sample with the lowest funding rate centers around the SPM system. This relation has the highest correlation rate (0.75). The next graph shows the link between the funding system and life expectancy at birth (Life_Expect_Q), where the sample with the lowest Life_Expect appears to be from the SPM system, which can be characterized as a system with a lower Expend_H. The highest Life_Expect at birth is associated with the NHS system. The correlation rate for this relation is equal to approximately 0.31. As for the perceived health status (Self_rep_H_Q), the most positive outputs can be attributed to the SPM system, and the least positive outputs can be attributed to the NHS system. The correlation rate for this is approximately 0.47. Very similar outputs are also shown in the graph assessing the health care index (HC_index_Q), where the SPM system has the least positive outputs, and the MI and NHS systems appear to be more effective. The correlation rate is 0.4. The relations in the previous graph should be interpreted with some caution, considering the outputs shown in Table 11.

## 4. Discussion

The fact that health capital plays a significant role in a country’s economic growth from a long-term perspective was confirmed in several studies [74,75]. Health of the population is an important determinant of economic development because a healthy population means higher productivity [27,76]. A healthy (laborable) population is the driving force of every economy. To increase economic performance, long-term economic development, or improve the quality of life in a country, adequate health care must be provided that reflects the opportunities for active product creation in the economy. For the improvement of national health and quality of life, some countries should actively look to optimize policy related to health expenditure, such as by enhancing the efficiency of health costs to promote sustainable economic development [77]. Investing in health care is an investment in human resources, which currently represent an indispensable part of product creation in every economy. For this reason, the relationship between the amount of health expenditure and outputs reflecting the effectiveness of health care in countries must be understood.

The results show the relationships between health expenditure and health care effectiveness represented by selected health care outputs (Life_expect, Self_rep_H, HC_index, AMId and DIAd) from a sample of OECD countries. Many authors [37,44] confirmed the positive relationship between health expenditure and health outcomes, but we offer a new perspective on the issue. We emphasize that when identifying this relationship, the system of financing the health care in the country must be considered as each system responds differently to the change in the amount of health expenditure, and no each system has to respond to a change in the amount of health expenditure.

By focusing on the links between health expenditure and life expectancy at birth in relation to the outcomes of both regression and correlation analysis, it is possible to speak of the existence of a certain relation (in the contextual analysis, the relationship did not appear only in the NHS system). These results partially confirm the results of the study of Bein et al. [37], who indicate the existence of a positive relationship. A very similar output can be seen in the relationship between health expenditure and perceived health status, where there is no significant relationship in SPM. On the other hand, Pierard [43] talks about the limited effect of expenditure. The results also reveal a partially unprovable relationship between health expenditure and the health care index. The regression analysis outputs showed the relationship in two of the three systems examined, but with a low coefficient of determination. The correlation analysis outputs complement this finding and show a significant output only for the NHS system. The other two cases show a questionable link, confirming an inconsistent connection [58]. The negative relationship between health expenditure and deaths from acute myocardial infarction can be stated in the case of the MI system, which indicates that a higher expenditure can predict a decrease in acute myocardial infarction death. These findings partially correlate with the claims made by Bradley et al. [48] and Moscone et al. [49]. The relationship between health expenditure and deaths from diabetes mellitus was also analyzed, and the expected negative relationship occurred only in the MI and SPM systems. Based on this and the findings of the study of Baudot et al. [56], it is possible to assume that an increased incidence of diabetes increases health expenditure, and an increase in health expenditure can result from a reduction in the number of deaths from diabetes mellitus.

In the first part of our analyses, the relationship between the health care financing system and selected variables that determine the effectiveness of health care was examined. A relationship has been identified, where the highest rate of association manifests itself in health expenditure, and the lowest manifests itself in deaths from acute myocardial infarction per 100,000 persons. Subsequently, a logistic regression analysis was applied in order to point out the increasing probability of the existence of a certain system under the influence of the selected variables of health care effectiveness in OECD countries. The probability that a given subject will have an MI system applied, compared to other systems, is significantly increased as health expenditure increases and the rate of diabetes mellitus deaths per 100,000 persons increases. However, the probability decreases as life expectancy at birth increases. These outputs tell us that the MI system is applied in countries where the life expectancy at birth is lower than in other countries, but where there is a higher expenditure on health care and more diabetes mellitus deaths, so there is some mismatch between health care funding and increasing health levels. The basic premise of economic theory is that if an input increases, the output should also increase. The NHS model is more likely to occur in countries with higher rates of a good or very good perceived health status and lower deaths from diabetes mellitus (Self_rep_H has a significant impact at 0.03, and DIAd is even more significant, i.e., the *p*-value is approximately 0.003). The probability that a given research entity will apply an SPM system, as opposed to other systems, is significantly increased when the health expenditure ratio decreases (a *p*-value of approximately 0.0001), when the perceived health status decreases, when the health care index decreases, and life expectancy at birth increases. Subsequently, a regression analysis was carried out to identify the impact of health expenditure on the selected variables that determine the effectiveness of health care. In the MI system, health expenditure had no significant impact on diabetes mellitus deaths; in the NHS, health expenditure had no significant impact on life expectancy at birth, deaths from acute myocardial infarction and diabetes mellitus deaths; and in the last system (SPM), the impact of health expenditure on the health care index had not been confirmed. When assessing the relations, where an impact has been confirmed, the degree of determination must also be taken into account. When assessing the impact of health expenditure on life expectancy at birth, the NHS funding model appears to be the most effective (*R^2^* = 0.57). NHS, with the MI system, appears to be the most optimal in terms of the perceived health status (MI: *R^2^* = 0.63; NHS: *R^2^* = 0.42). The MI system, together with NHS, seems to be the most effective system, even in terms of the health care index, but it is necessary to point out that it has a low determination coefficient, which is approximately 0.09 for MI and 0.13 for NHS. The largest difference between the systems was seen in diseases that are more typical in developed countries (which are considered OECD countries), meaning the mortality from acute myocardial infarction and diabetes mellitus. Here, the SPM seems to be the most effective. However, it should be pointed out that the coefficient of determination is lower. A correspondence analysis of the health care financing systems and quartile expressions of variables that determine the effectiveness of health care was then conducted. The linkage appears to be significant for all parameters, except death from acute myocardial infarction. When assessing health expenditure and health care financing systems, it can be said that the closest relationship is between the MI system and the highest health expenditure rate. As for life expectancy at birth, the closest relationship to the most positive outcomes is in the NHS system. The NHS also dominates the perceived health status. In the case of the health care index, the most positive values can be linked to the MI and NHS systems. The SPM shows a very close link with the least positive output. As for acute myocardial infarction, the outcomes are confusing, resulting in an insignificant interconnection. Concerning the last relationship, i.e., the interconnection of systems and the number of deaths from diabetes mellitus, it is clear that the most positive outcomes are in the NHS system, and the least positive are in the MI system.

In general, it is believed that it is appropriate to take the funding system into account in health-related analyses; otherwise, the results may be distorted. On the other hand, the findings of previous studies confirmed the significant impact of other socio-economic factors affecting the health of the population, such as social expenditure [38], income [1], accessibility to health care [4], innovation [59], education [8], the happiness and well-being of society [12] and unemployment [13]. 

## 5. Conclusions

The primary objective of the research in the present study was to determine the effectiveness of health care in classifying health care financing systems from a sample of OECD countries (between 2012 and 2017). As has been pointed out from several points of view, the individual systems do not show the same effectiveness rates. With great caution, it can be accepted that NHS and MI may be considered the most effective systems in general, and SPM seems to be less effective. It is also argued that, regarding effectiveness, there are differences between systems in terms of their predisposition to certain areas of health output. Where one system proves to be ineffective (or its effectiveness is questionable), another system(s) appears to be effective.

The limitations of the presented article lie in its focus on health expenditure and its impact on indicators representing the effectiveness of health care. In the future, the research will focus on verifying and deepening the outlined assumptions, i.e., to determine to what extent funding systems moderate the inputs and outputs of health systems. We plan to expand the portfolio of the analyzed variables to include expenditure items (inputs), health and effectiveness (outputs) and time evaluation (time series).

## Figures and Tables

**Table 1:** Gross health outputs of the Organization for Economic Cooperation and Development (OECD) countries (based on mean for the period 2012–2017).

Country	HC_Syste*m*	Expend_*H*	Life_expec*t*	Self_rep_*H*	HC_inde*x*	AMId	DIAd
Australia	NHS	9.03167	82.383	85.200	74.6717	36.620	19.260
Austria	MI	10.33950	81.417	69.733	80.2350	44.683	28.883
Belgium	MI	10.32900	81.133	74.417	79.4300	32.480	11.000
Canada	NHS	10.47967	81.817	88.583	70.6950	37.775	19.050
Czech Republic	MI	7.35200	78.717	60.650	67.8233	47.400	30.217
Denmark	NHS	10.18317	80.700	71.667	83.0300	26.725	20.650
Estonia	SPM	6.21217	77.450	52.383	73.0217	30.640	9.340
Finland	NHS	9.43467	81.317	68.500	73.4600	51.300	7.400
France	SPM	11.43650	82.467	67.483	82.0150	18.000	13.150
Germany	MI	11.02250	80.883	65.100	74.7783	45.680	20.520
Greece	SPM	8.27700	81.267	74.100	55.1667	44.520	10.140
Hungary	SPM	7.13433	75.767	57.483	52.4433	54.317	24.367
Chile	MI	8.01283	79.600	60.533	60.0033	51.440	37.160
Iceland	NHS	8.21933	82.583	76.380	65.7850	39.567	9.733
Ireland	NHS	8.75350	81.467	82.617	47.6450	55.600	14.375
Israel	MI	7.19067	82.217	81.567	80.2233	20.420	39.180
Italy	NHS	8.93800	82.867	69.267	66.2417	29.425	23.000
Japan	MI	10.84383	83.750	35.467	84.5583	18.020	6.360
Korea	SPM	6.96850	81.883	32.567	80.0100	26.360	27.240
Latvia	NHS	5.69433	74.400	45.883	66.0650	48.025	19.325
Lithuania	NHS	6.36650	74.617	44.133	68.4767	33.483	9.067
Luxembourg	SPM	5.62433	82.183	71.500	76.1100	29.340	13.600
Mexico	MI	5.68717	74.900	65.500	71.4750	134.480	153.200
Netherlands	MI	10.40267	81.567	76.117	70.5833	29.100	14.980
New Zealand	NHS	9.36783	81.567	89.183	76.0783	56.000	18.900
Norway	NHS	9.68067	82.183	77.667	76.5233	46.380	11.540
Poland	SPM	6.36900	77.533	58.183	58.1983	35.520	19.220
Portugal	NHS	9.07333	81.067	47.133	66.8917	32.520	31.420
Slovak Republic	MI	7.10600	76.817	65.917	62.0650	47.367	18.033
Slovenia	SPM	8.53717	80.850	64.533	65.5067	41.675	13.100
Spain	NHS	9.01150	83.117	72.917	74.4083	27.080	15.360
Switzerland	MI	11.71950	83.217	79.767	70.9450	22.660	12.620
Turkey	SPM	4.31633	77.450	68.183	69.6817	98.380	40.240
United Kingdom	NHS	9.47117	81.167	71.983	73.5050	35.940	8.280
United States	MI	16.66000	78.750	87.850	67.5983	36.320	24.460

Note: HC_System: Health Care System; Expend_H: Health expenditure in the percentage of GDP; Life_expect: Life expectancy at birth; Self_rep_H: Perceived health status; HC_index: Health Care Index; AMId: Deaths from acute myocardial infarction; DIAd: Deaths from diabetes mellitus; MI: Multiple insurance funds or companies; NHS: A national health system covering the country as a whole; SPM: A single health insurance fund (single-payer model).

**Table 2:** Descriptive statistics of quantitative variables in the classification of the health care financing systems.

HC_System		Expend_H	Life_expect	Self_rep_H	HC_index	AMId	DIAd
**Total**	***N***	210	209	208	198	169	169
	*M*	8.72132	80.320	67.399	70.3823	41.904	22.944
	*σ*	2.325369	2.6279	14.1619	9.55523	22.4545	24.6967
**NHS**	***N***	84	84	83	78	65	65
	*M*	8.83610	80.804	70.727	70.5773	39.165	15.714
	*σ*	1.372807	2.6960	14.8475	8.78958	9.9542	6.8487
**SPM**	***N***	54	54	54	48	44	44
	*M*	7.20837	79.650	60.713	66.9242	42.918	19.320
	*σ*	1.963008	2.5097	12.1298	11.45886	23.8262	9.8700
**MI**	***N***	72	71	71	72	60	60
	*M*	9.72214	80.256	68.594	72.4765	44.127	33.435
	*σ*	2.839983	2.5499	13.2052	8.36971	30.0071	37.8888

**Table 3:** Effect size of the health care financing systems (HC_System).

Dependent Variable	Expend_H	Life_expect	Self_rep_H	HC_index	AMId	DIAd
Value (*η^2^*)	0.173889	0.030625	0.082944	0.049729	0.009801	0.103041
Effect size	Medium	Small	Small	Small	Negligible	Small

Note: Negligible: *η^2^* < 0.02, small: *η^2^ =* 0.02–0.13, *η^2^ =* Mediun 0.13–0.26, Large *η^2^* > 0.26.

**Table 4:** Model fit statistic.

Test output	MCE			Deviance			Hoslem		
	Training	Testing	Difference	Null	Residual	Difference	X^2^	df	Sig.
Model 1 glm	0.25781	0.16129	0.09652	164.73000	113.37000	51.36000	13.4880	8	0.096
Model 2 glm	0.24219	0.09677	0.14541	170.35000	148.98000	21.37000	34.5900	8	3.175 × 10^5^
Model 3 glm	0.15625	0.12903	0.02722	146.11400	95.28500	50.82900	87.0820	8	1.776 × 10^15^

Note: Model 1 glm: MI (dependent variable), Expend_H, Life_expect, Self_rep_H, HC_index, AMId, DIAd (independent variables); Model 2 glm: NHS (dependent variable), Expend_H, Life_expect, Self_rep_H, HC_index, AMId, DIAd (independent variables); Model 3 glm: SPM (dependent variable), Expend_H, Life_expect, Self_rep_H, HC_index, AMId, DIAd (independent variables); df: Degrees of freedom; Sig.: asymptotic significance 2 sided.

**Table 5:** Models’ output.

Test output	Coefficients	Estimate	Std. Error	*z* Value	Sig.
**Model 1 (MI)**	Intercept	18.6161043	12.1882920	1.527	0.126668
	Expend_H	0.8688680	0.2297883	3.781	0.000156
	Life_expect	−0.3812744	0.1700773	−2.242	0.024976
	Self_rep_H	0.0005497	0.0182596	0.030	0.975986
	HC_index	0.0330331	0.0286964	1.151	0.249682
	AMId	−0.0379316	0.0240201	−1.579	0.114300
	DIAd	0.1360077	0.0315794	4.307	1.66 × 10^5^
**Model 2 (NHS)**	Intercept	−9.635026	8.634763	−1.116	0.26449
	Expend_H	−0.231847	0.140848	−1.646	0.09975
	Life_expect	0.112180	0.117958	0.951	0.34160
	Self_rep_H	0.040759	0.018538	2.199	0.02790
	HC_index	0.011036	0.021793	0.506	0.61259
	AMId	0.001823	0.015020	0.121	0.90341
	DIAd	−0.073380	0.025037	−2.931	0.00338
**Model 3 (SPM)**	Intercept	−14.70290	10.06726	−1.460	0.14416
	Expend_H	−1.00709	0.26322	−3.826	0.00013
	Life_expect	0.39186	0.15651	2.504	0.01229
	Self_rep_H	−0.06377	0.02635	−2.420	0.01552
	HC_index	−0.07570	0.03161	−2.395	0.01663
	AMId	0.01956	0.02249	0.870	0.38448
	DIAd	−0.06013	0.03085	−1.950	0.05123

**Table 6:** Regression assumptions, model selection and model representativeness.

System	Model	*N*	Model Variable	Unit Roots	Stationary	Outlier	Heteroscedasticityy	Regressioin Model	*R^2^*
**MI**	Model 1	71	Expend_H --> Life_expect	0,9749	<0.01	0.0510	0.2408	OLS	0.1497
	Model 2	71	Expend_H --> Self_rep_H	0.9998	<0.01	0.0045	0.3926	LTS	0.6276
	Model 3	72	Expend_H --> HC_index	0.6009	<0.01	0.0043	0.3850	LTS	0.0946
	Model 4	60	Expend_H --> AMId	0.9981	<0.01	0.0006 *	0.0171	OLS HC3	0.0382
	Model 5	60	Expend_H --> DIAd	0.9848	<0.01	0.0016 *	0.5756	OLS	0.1429
**NHS**	Model 6	84	Expend_H --> Life_expect	0.8630	<0.01	0.0269	0.0002	OLS HC3	0.5716
	Model 7	83	Expend_H --> Self_rep_H	0.9996	<0.01	0.0187	0.6007	OLS	0.4212
	Model 8	78	Expend_H --> HC_index	0.9585	<0.01	0.0000	0.1742	LTS	0.1332
	Model 9	65	Expend_H --> AMId	0.1152	<0.01	0.0096	0.0641	LTS	0.0410
	Model 10	65	Expend_H --> DIAd	0.8851	<0.01	0.0034	0.7392	LTS	0.0389
**NHS**	Model 11	54	Expend_H --> Life_expect	0.7907	<0.01	0.0337	0.0183	OLS HC3	0.2397
	Model 12	54	Expend_H --> Self_rep_H	1,0000	<0.01	0.0075	0.4002	LTS	0.2423
	Model 13	48	Expend_H --> HC_index	0.9942	<0.01	0.0732	0.3909	OLS	0.0508
	Model 14	44	Expend_H --> AMId	0.9997	<0.01	0.0066	0.0001	OLS HC3	0.2670
	Model 15	44	Expend_H --> DIAd	0.9718	<0.01	0.0357	0.0002	OLS HC3	0.2778

Note: *N*—number of observations entering subsequent analyzes after removal of missing values; Unit Roots—F test for individual effects (*p* Value); Stationary—Augmented Dickey Fuller Test (*p* Value); Outlier—Bonferroni Outlier Test; Heteroscedasticity—Breusch-Pagan Test. * Mexico 2012–2017 has been removed; OLS: Ordinary Least-Squares Regression; LTS: Least Trimmed Squares Robust Regression; OLS HC3: Heteroskedasticity-Consistent standard error estimators in Ordinary Least-Squares regression.

**Table 7:** Models’ output.

MI				NHS				SPM			
Model	B	Est.	Sig.	Model	B	Est	Sig.	Model	B	Est.	Sig.
Model 1 (Life_expect)	*α*	76.90	2.00 × 10^−^^16^	Model 6 (Life_expect)	*α*	67.68	2.20 × 10^−^^16^	Model 11 (Life_expect)	*α*	75.14	2.20 × 10^−^^16^
	*β_1_*	0.35	8.59 × 10^−^^4^		*β_1_*	1.48	3.17 × 10^−^^13^		*β_1_*	0.63	5.71 × 10^−^^8^
Model 2 (Self_rep_H)	*α*	48.44	2.00 × 10^−^^16^	Model 7 (Self_rep_H)	*α*	8.98	2.73 × 10^−^^1^	Model 12 (Self_rep_H)	*α*	41.02	3.17 × 10^−^^10^
	*β_1_*	2.32	2.86 × 10^−^^14^		*β_1_*	6.98	3.23 × 10^−^^11^		*β_1_*	2.67	3.27 × 10^−^^4^
Model 3 (HC_index)	*α*	64.17	2.00 × 10^−^^16^	Model 8 (HC_index)	*α*	57.31	2.00 × 10^−^^16^	Model 13 (HC_index)	*α*	57.43	6.20 × 10^−^^12^
	*β_1_*	0.93	9.09 × 10^−^^3^		*β_1_*	1.66	1.75 × 10^−^^3^		*β_1_*	1.29	1.24 × 10^−^^1^
sModel 4 (AMId)	*α*	45.18	5.84 × 10^−^^9^	Model 9 (AMId)	*α*	47.97	3.61 × 10^−^^9^	Model 14 (AMId)	*α*	89.39	5.82 × 10^−^^9^
	*β_1_*	−1.91	1.14 × 10^−^^1^		*β_1_*	−1.25	1.18 × 10^−^^1^		*β_1_*	−6.56	3.29 × 10^−^^4^
Model 5 (DIAd)	*α*	38.38	4.86 × 10^−^^9^	Model 10 (DIAd)	*α*	7.65	9.36 × 10^−^^2^	Model 15 (DIAd)	*α*	38.96	2.24 × 10^−^^7^
	*β_1_*	−1.56	4.43 × 10^−^^3^		*β_1_*	0.78	1.28 × 10^−^^1^		*β_1_*	−2.77	9.83 × 10^−^^4^

**Table 8:** Models’ prediction with mean health expenditure (8.72).

MI		NHS		SPM	
Model	Y	Model	Y	Model	Y
Model 1 (Life_expect)	79.91 **	Model 6 (Life_expect)	80.63	Model 11 (Life_expect)	80.60 *
Model 2 (Self_rep_H)	68.62	Model 7 (Self_rep_H)	69.87 ^+^	Model 12 (Self_rep_H)	64.30 *
Model 3 (HC_index)	72.24 **	Model 8 (HC_index)	71.74 **	Model 13 (HC_index)	-
Model 4 (AMId)	-	Model 9 (AMId)	-	Model 14 (AMId)	32.16 *
Model 5 (DIAd)	24.78 **	Model 10 (DIAd)	-	Model 15 (DIAd)	14.77 *

Note: * *R^2^* < 0.3; ** *R^2^* < 0.2; ^+^ non-significant constant.

**Table 9:** Corelation Matrix of analyzed variables total and in the classification of health care financing systems (HC_System).

Correlation *ρ*	MI						NHS					
	A	B	C	D	E	F	A	B	C	D	E	F
Expend_H		0.482	0.292	0.173	−0.431	−0.617		0.081	0.460	0.323	0.009	0.085
Life_expect	0.000		0.145	0.548	−0.806	−0.513	0.462		0.502	0.013	−0.188	0.022
Self_rep_H	0.013	0.230		0.050	−0.333	0.015	0.000	0.000		0.213	0.348	0.088
HC_index	0.147	0.000	0.681		−0.425	−0.246	0.004	0.908	0.063		0.008	0.015
AMId	0.001	0.000	0.009	0.001		0.586	0.944	0.133	0.005	0.950		−0.263
DIAd	0.000	0.000	0.910	0.058	0.000		0.499	0.860	0.489	0.911	0.034	
	**SPM**						**TOTAL**					
Expend_H		0.365	0.072	0.132	−0.248	−0.359		0.490	0.430	0.348	−0.272	−0.225
Life_expect	0.007		0.344	0.566	−0.662	−0.286	0.000		0.436	0.381	−0.553	−0.299
Self_rep_H	0.603	0.011		−0.165	0.201	−0.259	0.000	0.000		0.088	−0.004	−0.042
HC_index	0.370	0.000	0.261		−0.648	−0.033	0.000	0.000	0.222		−0.382	−0.089
AMId	0.105	0.000	0.190	0.000		0.361	0.000	0.000	0.963	0.000		0.317
DIAd	0.017	0.059	0.090	0.841	0.016		0.003	0.000	0.591	0.261	0.000	

Note: A -Expend_H; B - Life_expect; C - Self_rep_H; D - HC_index; E – AMId; F – DIAd.

**Table 10:** Analyzed variables in the country-quartile descriptions (based on mean).

Country	HC_System	Expend_H	Life_expect	Self_rep_H	HC_index	AMId	DIAd
Australia	NHS	Q2–Q3	Q3	Q3	Q2–Q3	Q1–Q2	Q2–Q3
Austria	MI	Q3	Q2–Q3	Q2–Q3	Q3	Q2–Q3	Q3
Belgium	MI	Q2–Q3	Q1–Q2	Q2–Q3	Q3	Q1–Q2	Q1
Canada	NHS	Q3	Q2–Q3	Q3	Q1–Q2	Q2–Q3	Q2–Q3
Czech_R	MI	Q1–Q2	Q1	Q1–Q2	Q1–Q2	Q2–Q3	Q3
Denmark	NHS	Q2–Q3	Q1–Q2	Q2–Q3	Q3	Q1	Q2–Q3
Estonia	SPM	Q1	Q1	Q1	Q2–Q3	Q1–Q2	Q1
Finland	NHS	Q2–Q3	Q2–Q3	Q1–Q2	Q2–Q3	Q3	Q1
France	SPM	Q3	Q3	Q1–Q2	Q3	Q1	Q1–Q2
Germany	MI	Q3	Q1–Q2	Q1–Q2	Q2–Q3	Q2–Q3	Q2–Q3
Greece	SPM	Q1–Q2	Q1–Q2	Q2–Q3	Q1	Q2–Q3	Q1
Hungary	SPM	Q1–Q2	Q1	Q1	Q1	Q3	Q2–Q3
Chile	MI	Q1–Q2	Q1–Q2	Q1	Q1	Q3	Q3
Iceland	NHS	Q1–Q2	Q3	Q2–Q3	Q1	Q2–Q3	Q1
Ireland	NHS	Q1–Q2	Q2–Q3	Q3	Q1	Q3	Q1–Q2
Israel	MI	Q1–Q2	Q3	Q3	Q3	Q1	Q3
Italy	NHS	Q1–Q2	Q3	Q1–Q2	Q1–Q2	Q1–Q2	Q2–Q3
Japan	MI	Q3	Q3	Q1	Q3	Q1	Q1
Korea	SPM	Q1	Q2–Q3	Q1	Q3	Q1	Q3
Latvia	NHS	Q1	Q1	Q1	Q1	Q3	Q2–Q3
Lithuania	NHS	Q1	Q1	Q1	Q1–Q2	Q1–Q2	Q1
Luxembourg	SPM	Q1	Q2-Q3	Q2–Q3	Q2–Q3	Q1	Q1–Q2
Mexico	MI	Q1	Q1	Q1–Q2	Q2–Q3	Q3	Q3
Netherlands	MI	Q3	Q2–Q3	Q2–Q3	Q1–Q2	Q1	Q1-Q2
New Zealand	NHS	Q2–Q3	Q2–Q3	Q3	Q2–Q3	Q3	Q1-Q2
Norway	NHS	Q2–Q3	Q2–Q3	Q3	Q3	Q2-Q3	Q1
Poland	SPM	Q1	Q1	Q1	Q1	Q1-Q2	Q2-Q3
Portugal	NHS	Q2–Q3	Q1–Q2	Q1	Q1–Q2	Q1-Q2	Q3
Slovak_R	MI	Q1	Q1	Q1–Q2	Q1	Q2-Q3	Q1-Q2
Slovenia	SPM	Q1–Q2	Q1–Q2	Q1–Q2	Q1	Q2–Q3	Q1–Q2
Spain	NHS	Q2–Q3	Q3	Q2–Q3	Q2–Q3	Q1	Q1–Q2
Switzerland	MI	Q3	Q3	Q3	Q1–Q2	Q1	Q1–Q2
Turkey	SPM	Q1	Q1	Q1–Q2	Q1–Q2	Q3	Q3
United Kingdom	NHS	Q2–Q3	Q1–Q2	Q2–Q3	Q2–Q3	Q1–Q2	Q1
United States	MI	Q3	Q1–Q2	Q3	Q1–Q2	Q1–Q2	Q2–Q3

**Table 11:** Correspondence Analysis – quantitative relevancy.

Test output		Expend_H	Life_expect	Self_rep_H	HC_index	AMId	DIAd
Eigenvalues	Variance (Dim 1)	0.3720	0.0740	0.1700	0.1150	0.0690	0.1370
	% of var. (Dim 1)	65.7930	81.8040	78.6160	71.2470	99.3840	97.5630
	Variance (Dim 2)	0.1930	0.0160	0.0460	0.0470	0.0000	0.0030
	% of var. (Dim 2)	34.2070	18.1960	21.3840	28.7530	0.6160	2.4370
*Χ^2^*	Value	118.7589	18.9051	45.0550	32.0890	11.7886	23.7699
	Sig.	2.97 × 10^−23^	4.33 × 10^−3^	4.56 × 10^−8^	1.57 × 10^−5^	6.69 × 10^-2^	5.76 × 10^−4^
Correlation (*π*)		0.7520	0.3008	0.4654	0.4026	0.2641	0.3750

## Abbreviations

AIS	Acute ischemic stroke
AMI	Acute myocardial infarction
AMId	Deaths from acute myocardial infarction
AMId_Q	Deaths from acute myocardial infarction - Quartile
DIAd	Deaths from diabetes mellitus
DIAd_Q	Deaths from diabetes mellitus - Quartile
Expend_H	Health expenditure in the percentage of GDP
Expend_H_Q	Expend_H - Quartile
GDP	Gross Domestic Product
HC_index	Health Care Index
HC_index_Q	HC_index - Quartile
HC_System	Health Care System
Life_expect	Average life expectancy at birth
Life_expect_Q	Life_expect - Quartile
MI	Multiple insurance funds or companies
NHS	A national health system covering the country as a whole
SPM	A single health insurance fund (single-payer model)
Self_rep_H	Perceived health status (surveyed 5-item scale - good and very good in percentage for all populations)
Self_rep_H_Q	Self_rep_H - Quartile
